# Autoimmune Pancreatocholangitis, Non-Autoimmune Pancreatitis and Primary Sclerosing Cholangitis: A Comparative Morphological and Immunological Analysis

**DOI:** 10.1371/journal.pone.0002539

**Published:** 2008-07-02

**Authors:** Irene Esposito, Diana Born, Frank Bergmann, Thomas Longerich, Thilo Welsch, Nathalia A. Giese, Markus W. Büchler, Jörg Kleeff, Helmut Friess, Peter Schirmacher

**Affiliations:** 1 Institute of Pathology, Technische Universität München, Munich, Germany; 2 Institute of Pathology, Helmholtz Zentrum München, Oberschleissheim, Germany; 3 Institute of Pathology, University of Heidelberg, Heidelberg, Germany; 4 Department of General Surgery, University of Heidelberg, Heidelberg, Germany; 5 Department of Surgery, Technische Universität München, Munich, Germany; Deutsches Krebsforschungszentrum, Germany

## Abstract

**Background:**

Autoimmune pancreatocholangitis (AIPC) is an emerging, not completely characterized disease. Aim of this study was the comprehensive evaluation of a series of AIPC patients, who were diagnosed and treated in a European institution between January 2003 and July 2006.

**Methodology/Principal Findings:**

Thirty-three patients with histologically confirmed AIPC were analyzed and compared to 20 patients with non-autoimmune chronic pancreatitis (CP) and 14 patients with primary sclerosing cholangitis (PSC). Clinical features and conventional histopathology were taken into account. Immunohistochemistry and real-time quantitative PCR were used for the characterization of the inflammatory infiltrate and the stromal reaction. AIPC was localized in the pancreatic head in 94% of the patients. Intra- and/or extrapancreatic biliary tract involvement was present in 64% of the cases. The number of infiltrating T-lymphocytes, macrophages and total plasma cells was significantly higher in AIPC than in CP (3-, 4- and 8-fold increase, respectively). The absolute number of IgG4-positive plasma cells was higher in AIPC than in CP and PSC (7-fold and 35-fold increase, respectively), but significance was only reached in comparison with PSC. CXCR5- and CXCL13-positive cells were almost exclusively detected in AIPC.

**Conclusions/Significance:**

AIPC is mainly a disease of the pancreatic head with possible extension into the periphery of the gland and/or into the biliary tract/gallbladder. The morphology of AIPC, as well as the immune- and stromal reaction is characteristic and comparable between cases with and without biliary tract involvement. Immunological markers (IgG4, CXCR5, CXCL13) can be of diagnostic relevance in specific settings.

## Introduction

Autoimmune pancreatitis (AIP) is a recently recognized clinicopathological entity, which was first described by Sarles in 1961 as a “chronic inflammatory sclerosis of the pancreas” of possible autoimmune pathogenesis associated with hypergammaglobulinemia.[1] The disease has been gaining new attention for the last two decades, and the term “autoimmune pancreatitis”, coined by Yoshida in 1995,[2] has only recently been widely accepted in the scientific literature.[3] Due to the possible involvement of the biliary tract, the term autoimmune pancreatocholangitis (AIPC) has been introduced.[4], [5] The main reasons for the rising interest in investigating AIPC reside in its increasing frequency, partly due to an increased awareness of the disease but also due to a potentially increased incidence in the last 20–30 years,[6], [7] its not yet clarified aetiology and pathogenesis and its still undefined clinical spectrum. Unfortunately, international consensus criteria for the diagnosis of AIPC are still missing.[8] The coexistence of AIPC with other autoimmune-related diseases, such as Sjögren's syndrome, inflammatory bowel diseases (IBD) and rheumathoid arthritis, the presence of immunologic abnormalities in subsets of patients (hypergammaglobulinemia, elevated serum IgG4 levels, presence of autoantibodies), and the association with a specific HLA-haplotype in the Japanese population, represent the main pieces of evidence of an autoimmune pathogenesis of the disease.[9], [10] Such evidence has been further supported by an animal model of an AIP-like form of chronic pancreatitis in neonatally thymectomized mice immunized with lactoferrin or carbonic anhydrase II.[11] Autoantibodies against lactoferrin or carbonic anhydrase isozymes are present in subgroups of AIPC patients [12], [13] and elevated carbonic anhydrase II autoantibodies are associated with increased serum IgG4 levels.[14] The clinical and serological features of AIPC are far from being uniform, so that a preoperative diagnosis is difficult and most patients are still subjected to probably unnecessary surgery.[15] Elevated serum levels of IgG4 have been reported to be of diagnostic value in some series,[16], [17] whereas other groups have shown that a mild (≤2-fold) elevation of IgG4 levels can also occur in other settings, such as non-autoimmune chronic pancreatitis and pancreatic cancer.[18] The immunohistochemical evaluation of IgG4-positive plasma cells in pancreatic tissues has been proposed as an alternative marker of AIPC.[19] However, the use of this parameter in biopsy material is impaired by the patchy distribution of IgG4-positive cells in AIPC.[6]

This complex and controversial scenario renders the analysis of large series of histologically confirmed AIPC necessary, in order to accumulate further data that can improve and extend the present knowledge about this challenging disease.

In this single institutional study, a collective of 33 patients with histologically proven AIPC is presented and characterized from the clinical and pathological point of view, with particular attention to the biliary tract involvement and to the analysis of the inflammatory response and the stromal reaction. The results are compared with those obtained in two control groups, consisting of histologically confirmed non-autoimmune chronic pancreatitis and primary sclerosing cholangitis. In order to define distinct and discriminative features of AIPC, the number and distribution of B and T lymphocytes, macrophages and plasma cells, including the subclass of IgG4-positive plasma cells, were analyzed. Moreover, the expression of CXCL13 (BCA-1, B-cell attracting chemokine 1) and CXCR5 (BLR1, Burkitt lymphoma receptor-1) was examined, aiming at identifying further AIPC-specific tissue markers possibly related to the autoimmune pathogenesis of this disorder. CXCL13 and CXCR5 are in fact involved in normal lymphocyte trafficking and homing in secondary lymphoid organs and are highly expressed in various autoimmune diseases characterized by the occurrence of ectopic lymphoid follicles [20]–[23], which are also a prominent feature of AIPC. The peculiar morphological aspect of the AIPC-associated fibrosis, also termed “inflammatory storiform fibrosis” [24] due to the presence of inflammatory cells and plump myofibroblasts within irregular whorls of collagen bundles, prompted the further characterization of the stroma composition, in an attempt to provide an insight into the molecular basis of different patterns of pancreatic fibrosis.

## Materials and Methods

### Patients and tissue collection

Thirty-one cases of AIPC were diagnosed among 320 patients with histologically proven chronic pancreatitis operated on between January 2003 and July 2006 at the Department of Surgery of the University of Heidelberg, thus representing 9.7 % of all CP-patients, who received surgical therapy at this institution. Twenty-four AIPC patients (73%) underwent surgical exploration/resection under the suspicion of a malignant pancreatic tumour. Two of them (patients 31 and 33, see Table 1) showed involvement of the extrahepatic biliary tree with morphological similarities to autoimmune pancreatitis and were therefore included in this series. AIPC-patients 2–9, 11–14, 16–17, 25, 30 and 32 had been included in a previously published series focused on clinical aspects of the disease.[15] The group of AIPC patients was compared to a cohort of 20 patients (16 males, 4 females) affected by non-autoimmune CP (30% with confirmed chronic alcohol abuse) as well as to a group of 14 patients (10 males, 4 females) affected by PSC. Of the 14 PSC patients, 11 (78%) were subjected to liver transplantation due to end-stage disease. Two patients with mostly peripheral disease were treated either by duodenum-preserving pancreatic head resection (DPPHR) with cholecystectomy or by cholecystectomy alone. Eleven PSC patients (78%) were affected by ulcerative colitis and 1 patient (7%) by Crohn's disease. Formalin-fixed paraffin embedded tissue samples were available from all patients. Snap-frozen tissues preserved at −80°C for RNA extraction were available from 7 AIPC cases (pancreas), 5 non-autoimmune CP cases (pancreas) and 5 PSC cases (liver).

**Table 1:** Clinicopathological characteristics of AIPC patients.

Patient	Sex	Age	Clinical presentation	Preoperative diagnosis	Surgical treatment	Site of disease	Serum IgG4 (g/l)	Follow-up/associated diseases	Grading	Activity
1	M	48	jaundice	tumour	PD	Intrapancreatic	-	ok	2	low
2	M	44	pain	tumour	ppPD	Intrapancreatic	-	ok	4	low
3	M	33	pain	pancreatitis	DPPHR	Intrapancreatic	-	ok	3	high
4	M	34	pain	tumour	PD	Intrapancreatic	0.39	-	2	low
5	F	50	pain	tumour	ppPD	Intrapancreatic	-	ok	3	high
6	M	23	pain	unclear	Segmental resection	Intrapancreatic	-	ok	3	low
7	M	33	pain	unclear	DPPHR	Intrapancreatic	0.4	ok	3	low
8	M	37	jaundice	tumour	ppPD	Intrapancreatic	0.2	ok	4	low
9	M	64	pain, jaundice	unclear	PD	Intrapancreatic	-	AIP rec.	3	low
10	F	40	pain	tumour	ppPD	Intrapancreatic	-	SLE	2	high
11	M	32	pain	tumour	ppPD	Intrapancreatic	-	-	3	high
12	M	16	pain/jaundice	tumour	ppPD	Intrapancreatic	-	cholangitis	3	low
13	M	33	pain/jaundice	tumour	ppPD	intrapancreatic	10.9§	-	4	low
14	M	22	pain/jaundice	tumour	ppPD	intrapancreatic	0.31	IBD (UC)	3	high
15	M	70	pain/jaundice	tumour	ppPD	intrapancreatic	1.7	ok	4	low
16	M	66	pain	tumour	ppPD	intrapancreatic	-	ok	3	low
17	M	58	pain/jaundice	tumour	ppPD	intrapancreatic	-	ok	4	low
18	M	70	jaundice	tumour	ppPD	intrapancreatic	-	ok	4	high
19	M	40	pain	pancreatitis	ppPD	intrapancreatic	-	ok	3	low
20	F	26	pain	tumour	ppPD	intrapancreatic	0.4	ok	2	low
21	M	53	jaundice	tumour	DPPHR	intrapancreatic	-	-	3	low
22	M	78	jaundice	cystic tumour	ppPD	intrapancreatic	-	-	3	low
23	M	21	jaundice	pancreatitis	ppPD	intrapancreatic	-	-	3	low
24	M	48	jaundice	pancreatitis	PD	intrapancreatic	1.9	-	4	low
25	F	39	pain	unclear	ppPD	intrapancreatic	-	ok	2	high
26	M	42	pain	tumour	ppPD	intrapancreatic	-	IBD (UC)#	3	low
27	M	65	pain, jaundice	tumour	ppPD	intra/extrapancreatic (cystic duct)	-	ok	4	low
28	M	64	pain	cystic tumour	ppPD	intrapancreatic	-	ok	2	high
29	F	49	pain	tumour	Left subtotal pancreatectomy	intrapancreatic	0.51	ok	4	low
30	M	77	jaundice	tumour	ppPD	intra/extrapancreatic (GB)	-	ok	2	high
31	M	64	jaundice	tumour	Left hemihepatectomy	extrapancreatic (liver, EBD)	-	dead	*	*
32	M	54	pain	pancreatitis	ppPD	intra/extrapancreatic (GB)	-	ok	4	low
33	M	36	pain	tumour	EBD resection, bilio-digestive anastomosis	extrapancreatic (EBD, IBD)	-	cholangitis	*	*

**PD**: pancreaticoduodenectomy; **ppPD**: pylorus-preserving pancreaticoduodenectomy; **DPPHR**: duodenum-preserving pancreatic head resection; **GB**: gallbladder; **EBD**: extrahepatic bile duct; **IBD**: intrahepatic bile duct.

**ok**: no evidence of recurrent disease/associated disease; no disease/operation-associated symptoms.

**-**: not available; n: normal; **SLE**: systemic lupus erythematosus; **IBD**: inflammatory bowel disease; **UC**: ulcerative colitis.

# UC known pre-operatively.

* grading and activity not determined since no pancreatic tissue available.

§ normal range: 0.052–1.25 g/l.

The study was approved by the ethics committee of the University of Heidelberg, Germany, thus written informed consent was obtained from all patients.

### Clinical features and follow-up

Clinical and follow-up information was obtained from the clinical records and in some cases by direct contact with the patient. Together with the demographic characteristics, the following features were recorded: history of CP in the family, personal history of alcohol abuse, gallstones, autoimmune-related diseases (in particular Sjögren Syndrome, ulcerative colitis, Crohn's disease), main symptoms, and preoperative laboratory values. Data about serum immunoglobulin levels were available in 9 patients. In the follow-up analysis, the development of known AIP-related diseases (for example, sialadenitis, inflammatory bowel disease, tubulonephritis, retroperitoneal fibrosis) was also taken into account.

### Histology, immunohistochemistry and morphometry

Routine histological examination of formalin-fixed, paraffin-embedded tissue sections stained with hematoxylin and eosin (H&E) obtained from pancreatic specimens was performed by two experienced pathologists (IE and FB) with special training in pancreatic pathology. The diagnosis and grading of severity and activity of AIP were assessed according to previously described criteria.[24] All the non-autoimmune CP specimens were scored according to a previously reported system.[25] All PSC specimens were retrieved from the archives of the Institute of Pathology of the University of Heidelberg and comprehensively reviewed for the purposes of the present study.

Immunohistochemistry was performed according to the streptavidin-biotin method using diaminobenzidine as a chromogen, as previously described.[26] For the CXCL13, CXCR5 and α-SMA staining, the Envision^TM^ Detection-System (DakoCytomation, Hamburg, Germany) was used. All other reagents were from KPL (Kirkegaard and Perry Laboratories, Gaithersburg, MD, USA). The antibodies used in this study and the respective protocols are shown in Table 2. Morphometric analysis of the inflammatory infiltrate was performed by measuring the whole tissue area of each section with the computer program Image-Pro® Plus version 5.1 (IPWIN32.exe-Software, Weiss Imaging and Solutions, Bergkirchen/Günding, Germany) and counting the stained cells with the aid of an image analyser (Olympus Soft Imaging Solutions, Münster, Germany). The intensity of immunostaining of the stromal reaction was semiquantitavely evaluated (absent = 0; mild = +; moderate = ++; strong = +++).

**Table 2:** Antibodies and protocols for immunohistochemistry.

Antigen/Clone	Cell type/Site of expression†	Company	Dilution	Antigen retrieval	Detection system
IgG_4_/HP 6025	Plasma cells	Zymed Laboratories Inc, San Francisco/USA	1∶1000	1 mM EDTA (pH = 8.0)	Streptavidin-Peroxidase
Syndecan/MI15	Plasma cells	Dako-Cytomation	1∶25	Citrate buffer (pH = 6.0)	Streptavidin-Peroxidase
CD5/54/F6	T lymphocytes	Dako-Cytomation	1∶25	Tris-EDTA-Buffer (pH = 9.0)	Streptavidin-Peroxidase
CD20/L26	B lymphocytes	Dako-Cytomation	1∶100	Dako Target Retrieval Solution (pH = 6.1)	Streptavidin-Peroxidase
CD68/KP1	Macrophages	Dako-Cytomation	1∶1000	Citrate Buffer (pH = 6.0)	Streptavidin-Peroxidase
CXCL13/53610	Follicular dendritic cells	R&D Systems, Minneapolis/USA	1∶20	Dako Target Retrieval (pH = 9.0) solution	Envision
	B lymphocytes				
	Macrophages				
CXCR5	B lymphocytes	Abcam, Cambridge/UK	1∶100	Citrate buffer (pH = 6.0)	Envision
	Subsets of T lymphocytes				
SMA/1A4	Activated stellate cells	Dako-Cytomation	1∶150	None	Envision
	Smooth muscle cells				
TenascinC/BC-24	Activated stellate cells	Abcam, Cambridge/UK	1∶4000	Pronase 1∶10	Streptavidin-Peroxidase
	Extracellular matrix				
Collagen I/COL1	Extracellular matrix	Abcam, Cambridge/UK	1∶50	None	Streptavidin-Peroxidase
Collagen III	Extracellular matrix	SantaCruz Biotechnology Inc., USA	1∶500	None	Streptavidin-Peroxidase
Collagen V	Activated stellate cells	SantaCruz Biotechnology Inc., USA	1∶200	None	Streptavidin-Peroxidase
	Extracellular matrix				
Collagen VI	Extracellular matrix	SantaCruz Biotechnology Inc., USA	1∶200	None	Streptavidin-Peroxidase

† only the expression sites relevant to the present study are indicated.

### Real-time quantitative PCR

All reagents for mRNA extraction and cDNA synthesis were from Roche Applied Science (Mannheim, Germany). mRNA was isolated with the MagNA Pure LC Instrument using the Isolation Kit II. cDNA was synthetized using the First Strand cDNA-Synthesis-Kit for RT-PCR (AMV). The LightCycler FastStart DNA SYBR green kit was employed for the PCR analysis. The number of specific transcripts was calculated for the standard curve and normalized to two houskeeping genes CPB (Cyclophilin B) und HPRT (Hypoxanthin Phosphoribosyltransferase). All primers were from Search LC (Mannheim, Germany).

### Statistical analysis

Statistical analysis was performed with the software program GraphPad Prism4 (GraphPad Software, San Diego, CA, USA). Data are expressed as mean (±SEM), unless otherwise specified. The Student's t-test or the non-parametric Mann-Whitney U-test was used for comparisons, where appropriate. Associations between clinical and pathologic features were assessed by the Fisher's exact test or the χ^2^ test. Significance was defined as p≤0.05.

## Results

### Clinical and morphological characterization

#### Clinical data and follow-up

Most AIPC-patients (84%) were middle-aged men (44±31 years old, median±IQR). Obstructive jaundice and/or abdominal/back pain were the most common pre-operative symptoms and elevated values of pancreatic enzymes and cholestasis parameters were the most frequent laboratory abnormalities. The IgG4 serum levels were measured in 9 patients and a 4.4-fold increase was found in 1 of them (12%). Three patients (9%) had an extrapancreatobiliary autoimmune-related disease (2 cases with ulcerative colitis, 1 case with systemic lupus erythematosus). The detailed demographic and clinical characteristics of AIPC-patients are shown in Table 1. Table 3 shows a comparison of the main clinical and laboratory findings in the different disease groups. When the AIPC-cases with and without extrapancreatic biliary tract involvement (AIPC extrapancreatic and AIPC intrapancreatic, respectively) were considered separately, the results of the comparison of the entire AIPC group with CP and PSC were confirmed (Table 3). No significant differences in the comparison between AIPC intra- and extrapancreatic were found (not shown).

**Table 3:** Overview and comparison of the main clinical and laboratory findings in AIPC, CP, and PSC.

	AIPC total	AIPC intra-pancreatic	AIPC extra-pancreatic	CP	PSC	***AIPC total vs CP***	***AIPC total vs PSC***	***AIPC intra-pancreatic vs CP***	***AIPC extra-pancreatic vs CP***	***AIPC extra-pancreatic vs PSC***
**Male∶female**	28∶5	23∶5	5∶0	16∶4	10∶4	*ns*	*ns*	*ns*	*ns*	*ns*
**Age (median±IQR)**	44±31	41±22.5	64±26	48.5±14	38±21	*ns*	*ns*	*ns*	*ns (0.08)*	***0.02***
**Type of operation** * ** (DPPHR∶ppPD∶PD∶O)**	3∶22∶4∶4	3∶19∶4∶2	0∶3∶0∶2	8∶8∶3∶1	-	***0.04***	*-*	*-*	*-*	*-*
**Tumour suspicion** *	24/33 (73%)	20/28 (71%)	4/5 (80%)	3/20 (15%)	-	***0.0007***	*-*	*-*	*-*	*-*
**Amylase (U/l)**	74±15.3	77±17.9	68±17.6	99±19.3	54±8.8	*ns*	*ns*	*ns*	*ns*	*ns*
**Lipase (U/l)**	195±38	194±48	198±68	81±20.4	109±37	***0.008***	*ns*	***0.01***	***0.03***	*ns*
**Total bilirubin (mg/l)**	2.1±0.5	2.1±0.7	1.7±0.5	0.6±0.1	9.5±3.5	***0.002***	***0.01***	***0.03***	***0.0009***	*ns* §
**AP (U/l)**	189±34	196±40	164±60	84±8.4	461±116	***0.01***	***0.001***	***0.01***	***0.02***	*ns* §
**GGT (U/l)**	220±47	211±53	257±110	110±43.7	123±41	***0.02***	*ns*	*ns (0.08)*	***0.01***	*ns (0.07)*

**DPPHR**: duodenum-preserving pancreatic head resection; **ppPD**: pylorus preserving pancreaticoduodenectomy; **PD**: pancreaticoduodenectomy; **O**: others; **AIPC total**: all AIPC cases are included; **AIPC intrapancreatic**: cases with no extension into the extrapancreatic biliary tract; **AIPC extrapancreatic**: cases with extension into the extrapancreatic biliary tract.

* for these parameters, only AIPC total and CP groups are compared.

§ probably due to low sample number in the AIPC extrapancreatic group.

Data are expressed as mean±SEM, unless otherwise specified.

#### Morphology

##### Pancreas

**Figure 1:**
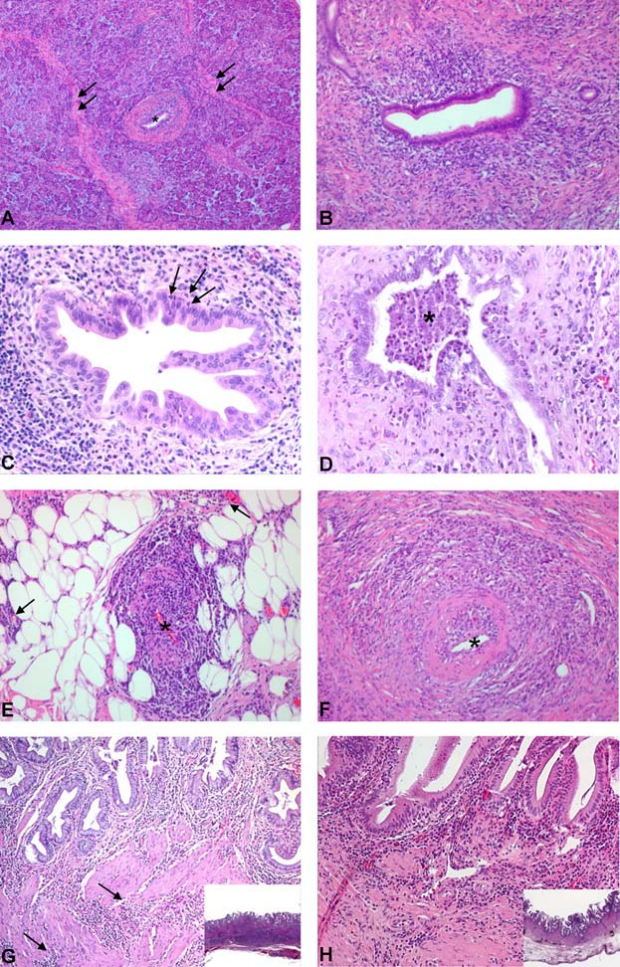
Histopathological features of AIPC. (A) Overview of pancreatic parenchyma with typical AIPC changes. In the center, a duct (*) with periductal mononuclear infiltration and fibrosis. Interlobular fibrosis is shown (arrows). (B) Interlobular duct with moderate periductal mononuclear infiltration, fibrosis and beginning stenosis of the lumen. (C) Higher magnification of an interlobular duct with early granulocytic epithelial lesion (GEL). Note the few neutrophils that infiltrate through the basal membrane into the epithelium (arrows). (D) Advanced GEL with abscess formation (*) in the lumen of an interlobular duct. (E) Lymphoplasmocytic inflammation of the wall of a small peripancreatic vein (*). Note the extension of the inflammatory infiltrate into the peripancreatic fat (arrows). (F) Lymphoplasmocytic inflammation of the wall of a small-sized intrapancreatic artery (*). (G) Involvement of the gallbladder in a case of AIPC. In the mucosa, a dense lymphoplasmacytic infiltrate below an almost intact epithelium. Note the thickening of the wall, due to the fibrosis and the extension of the inflammation into the deeper layers (arrows, inset). (H) Involvement of the gallbladder in a case of primary sclerosing cholangitis. Here, also a predominant lymphoplasmacytic inflammation with focal erosions of the epithelium and only superficial fibrosis. The deeper portions of the gallbladder wall are intact (inset).

Typical features of AIP were present in all cases in which pancreatic tissue was available for the macroscopical and histological analysis (n = 31) (Figure 1, A–F). No patient had calculi or calcifications of the pancreatic parenchyma, irregular duct dilatation or anatomical anomalies (e.g. pancreas divisum), which are typically associated with chronic pancreatitis. They rather showed a diffuse enlargement of the pancreas, mostly (n = 29, 94%) localized in the pancreatic head. Patient 28 had in addition an inflammatory pseudocyst of the pancreatic head, a rare finding in AIP. This case has been published previously.[27] Regarding the histological grade of severity of the disease, seven patients (23%) had a score 2, fourteen patients (45%) a score 3, and ten patients (32%) a score 4. The activity of the inflammatory process was scored as “low” ( = absence of/focal granulocytic epithelial lesions, GEL; no significant granulocytic infiltrate in the acinar parenchyma) in 22 patients (71%) and as “high” ( = multifocal GEL with intraductal abscess formation; granulocytic infiltrate in the acinar parenchyma) in 9 patients (29%) (Table 1). Grade and activity did not correlate. Cases with high activity had more frequently elevated serum levels of pancreatic enzymes (amylase and /or lipase) than cases with low activity, but the difference was not statistically significant.

All 20 CP patients revealed the typical features of non-autoimmune CP, with mainly interlobular or diffuse fibrosis, atrophy of the acinar parenchyma, dilatation of the pancreatic ducts (18 cases, 90%), calcifications (9 cases, 45%), necroses (12 cases, 60%), and pseudocysts (5 patients, 25%). According to a previously established scoring system, the severity of the inflammatory process was mild (score 1) in 6 patients (30%), moderate (score 2) in 10 patients (50%), and severe (score 3) in 4 cases (20%). Morphological features of AIP, such as periductal inflammation, GEL, venulitis, and/or arteriitis were absent in all cases. Scattered lymph follicles within and around the pancreatic parenchyma were observed in 13 cases (65%).

##### Biliary tree

A typical morphological finding in AIPC was the involvement of the intrapancreatic bile duct, which was severely affected by the inflammatory process with stenosis of the lumen in 16 cases (48%). In addition, three patients (patients 27, 30 and 32) showed an extension of the disease into the extrapancreatic biliary tree, with involvement of the common bile duct/cystic duct/gallbladder. Furthermore, in patients 31 and 33 the histological analysis of the extra- and/or intrahepatic bile duct showed typical AIPC-like changes, so that a total of 5 patients (15%) showed extrapancreatic extension of the disease into the biliary tree. The morphological features of the biliary tract involvement were comparable to the typical intrapancreatic findings, i.e. a periductal, mostly mononuclear cell infiltrate rich in plasma cells with only focal detachment of the overlying epithelium and a “storiform” fibrosis of all layers of the wall with stenosis of the lumen (Figure 1G). In case 31, a left hemihepatectomy was performed under the suspicion of liver metastases. The histopathological examination of the liver revealed parenchymal atrophy with enlarged portal tracts showing periductal chronic inflammation and obliteration of the bile ducts. No laboratory or conventional histologic parameter was predictive of the extrapancreatic bile duct involvement in the cases with pancreatic disease.

PSC patients that underwent liver transplantation displayed by histological analysis the typical features of biliary cirrhosis. In two patients with peripheral disease, the gallbladder showed periglandular dense lymphoplasmocytic infiltration with focal epithelial detachment and periductal fibrosis, which however did not have the dense and storiform pattern observed in AIPC cases with biliary tract involvement and which was limited to the upper third of the organ's wall (Figure 1H).

### Comparative analysis of the inflammatory and stromal reaction

#### Inflammatory infiltrate

**Figure 2:**
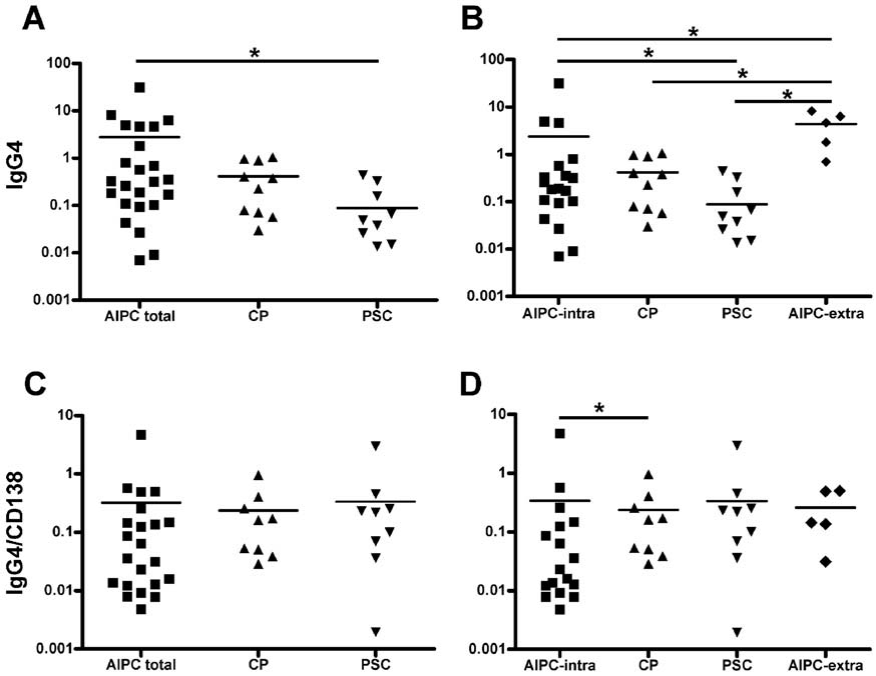
Quantitative evaluation of the IgG4-positive cells and of the IgG4/plasma cell ratio. (A) Total number of IgG4-positive cells and (C) IgG4/CD138 ratio in the disease groups of AIPC total, CP and PSC. (B) Total number of IgG4-positive cells and (D) IgG4/CD138 ratio when the AIPC cases are subclassified in those with and without extrapancreatic biliary tract involvement (AIPC extrapancreatic and AIPC intrapancreatic, respectively). Y-axis indicates the number of positive cells/mm^2^ in a logarithmic scale for better representation. Horizontal bars represent mean. * indicates statistical significance (p≤0.05).

The morphological characterization of the inflammatory infiltrate showed a clear-cut predominance of mononuclear cells. However, the number and distribution of the different cell types, as shown by the immunohistochemical analyses, were quite different. In detail, the number of B- and T-lymphocytes, macrophages and in particular of plasma cells was higher in AIPC with and without biliary tract involvement than in CP and PSC. The absolute number of IgG4-positive plasma cells was higher in AIPC (both groups) compared to CP and PSC. The group with the highest number of IgG4-positive plasma cells was that of AIPC with involvement of the extrapancreatic biliary tract. However, when the number of IgG4-positive plasma cells was normalized to the total number of plasma cells, as determined by CD138 staining, no significant differences between the different groups were found (Figure 2), indicating that there was no selective increase of IgG4-positive plasma cells compared to the other subclasses. Only in the group of AIPC with intrapancreatic involvement there was a slightly significant increase in the IgG4-CD138 ratio in comparison with non-autoimmune CP (p = 0.04). Regarding the localization, the inflammatory cells were present both within and outside the pancreas (in the peripancreatic adipose tissue) in AIPC, whereas they were mostly intrapancreatic in CP. In AIPC, B- and T-lymphocytes and plasma cells often displayed a periductal localization, which was not observed in CP. In PSC, the inflammatory cells were distributed in a diffuse fashion or in small clusters in the cirrhotic septa or in the mucosal layer of the gallbladder.

**Figure 3:**
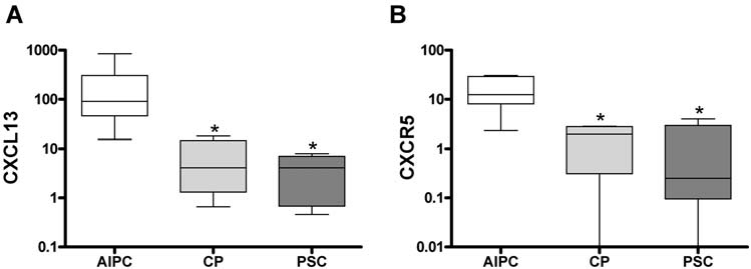
Real-time quantitative PCR analysis of CXCR5 and CXCL13. Real-time quantitative RT-PCR analysis of pancreatic tissues showing significantly higher levels of CXCL13 (A) and CXCR5 (B) mRNA in AIPC than in CP and PSC. RNA input was normalized to the average expression of the two housekeeping genes HPRT and cyclophilin B as described in *Methods*. Y-axis indicates the relative amount of mRNA in a logarithmic scale for better representation. Box and whiskers blot with 10–90 percentile are shown. * indicates statistical significance (p≤0.05).

The number of CXCR5-positive cells was significantly higher in AIPC (both subgroups) than in the other disease groups, with a mostly periductal and diffuse parenchymal localization. CXCL13-positive cells were only found in the AIPC groups in a periductal and diffuse parenchymal localization. To confirm the overexpression of CXCR5 and CXCL13 in AIPC, real-time quantitative PCR was additionally performed. Median CXCR5-mRNA levels in AIPC showed a 4.1-fold increase in comparison with CP (p = 0.02) and a 32-fold increase in comparison with PSC (p = 0.005). Median CXCL13-mRNA levels in AIPC were also significantly higher than in the other groups, with a 21-fold increase compared to CP (p = 0.01) and a 22-fold increase compared to PSC (p = 0.002) (Figure 3).

**Figure 4:**
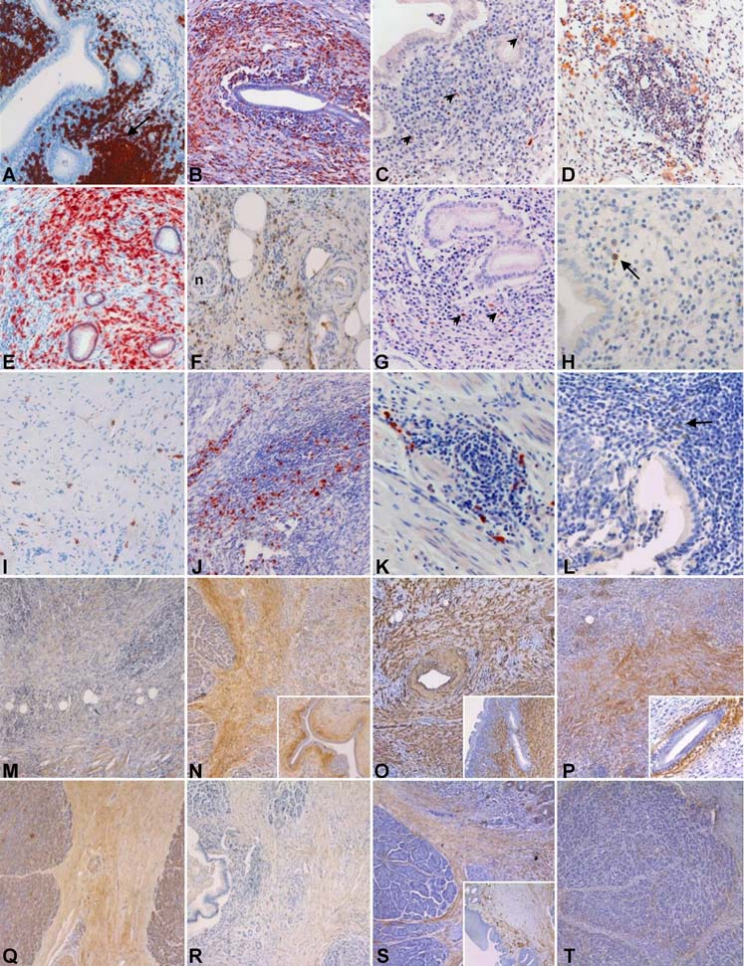
Immunohistochemical characterization of AIPC, CP and PSC. (A–H) Immunohistochemical characterization of the inflammatory infiltrate of AIPC. (A) CD20-positivity of B-lymphocytes in periductal localization and with tendency to form lymph follicles (arrow). (B) CD5-positive T-lymphocytes with diffuse arrangement around an interlobular duct. (C) Scattered CXCR5-positive cells (arrowheads) around the main pancreatic duct. (D) CXCL13-positive cells arranged in small clusters in the pancreatic tissue. (E) Numerous CD138-positive plasma cells in a diffuse arrangement in the pancreatic parenchyma. (F–G) IgG4-positive cells diffusely distributed in the peripancreatic adipose tissue (F) and in a periductal localization (G). (H) Isolated IgG4-positive cells (arrow) below the epithelium of the gallbladder in a case of AIPC with extrapancreatic biliary involvement. (I–K) Immunohistochemical characterization of the inflammatory infiltrate in CP. (I) A few CD138-positive plasma cells in a diffuse arrangement in pancreatic scar tissue. (J–K) IgG4-positive cells in large (J) and small (K) clusters in the pancreatic parenchyma. (L) Isolated IgG4-positive cells (arrow) in the gallbladder of a patient affected by PSC. (M–P) Immunohistochemical characterization of the stromal reaction in AIPC. (M) Low-power view of pancreatic tissue shows a weak (+) to moderate (++) interstitial staining for collagen I. (N) Moderate (++) to strong (+++) interlobular and periductal (inset) collagen V staining. (O) Strong (+++) diffuse and periductal (inset) α-smooth muscle actin (α-SMA) staining. (P) Diffuse interlobular and strong (+++) periductal (inset) positivity for Tenascin C. (Q–T) Immunohistochemical characterization of the stromal reaction in CP. (Q) Moderate (++) interlobular collagen I staining. (R) Very weak (−/+) interlobular collagen V staining. (S) Moderate (++) positivity for α-SMA in the interlobular connective tissue, without periductal arrangement (inset, here notice the extensive squamous cell metaplasia of the duct epithelium). (T) Moderate (++) staining for Tenascin C at the interface between the pancreatic lobules and the interlobular connective tissue.

No significant differences in cell count and distribution were observed when AIPC with and without extrapancreatic extension were compared to each other (not shown). The immunohistochemical characterization of the inflammatory infiltrate is shown in Table 4 and exemplarily illustrated in Figure 4 (panels A–L).

**Table 4:**
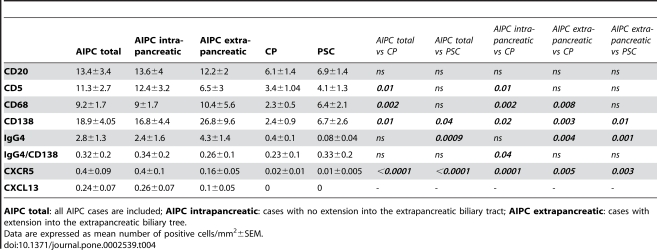
Overview and comparison of the immunohistochemical analysis of the inflammatory infiltrate in AIPC, CP and PSC.

**AIPC total**: all AIPC cases are included; **AIPC intrapancreatic**: cases with no extension into the extrapancreatic biliary tract; **AIPC extrapancreatic**: cases with extension into the extrapancreatic biliary tree.

Data are expressed as mean number of positive cells/mm^2^±SEM.

#### Stromal reaction

The morphological analysis of the stromal reaction revealed increased extracellular matrix deposition in all different disease groups. Collagen I, III, V and VI were all expressed in the AIPC and CP group, although with different patterns and intensities. Collagen I staining was mostly weak in the AIPC group, where it showed a preferentially interlobular localization. In CP, collagen I staining was generally more intense, in particular in score 3 cases. Collagen III staining was moderate to intense in the AIPC group and it showed a mostly periductal localization. In CP, intensity and pattern of expression of collagen III were overlapping with those of collagen I. Collagen V immunostaining revealed a moderate extracellular expression as well as a moderate to strong expression in stromal fibroblasts in the AIPC group, with a preferential interlobular and periductal localization. In the CP group, a moderate, mostly extracellular interlobular collagen V staining was found. Collagen VI was also strongly expressed in the AIPC group (interlobular and periductal), whereas it was relatively weak in CP tissues. PSC tissues displayed a diffuse, weak to moderate positivity for collagen I and VI in the cirrhotic septa. No significant staining for collagen III or V was found in PSC.

Cytoplasmic α-SMA expression in stromal fibroblasts and extracellular tenascin C expression displayed overlapping localizations. In all three groups, the intensity of the staining was moderate to intense. In AIPC, the staining was mostly periductal and inter-/intralobular, consisting of broad stained areas of connective tissues between the acinar lobuli. In CP, the staining was also periductal and interlobular, but less extensive and often localized to a thin band of connective tissue just around the acinar lobuli. Finally, in PSC, the staining was diffuse in the cirrhotic septa, often with a strongly positive band just around the cirrhotic nodules. The immunohistochemical characterization of the stromal reaction is exemplarily shown in Figure 4 (panels M–T). Quantitative PCR analysis revealed higher Tenascin C and α-SMA mRNA levels in AIPC compared to the other diseases. In detail, Tenascin C m-RNA levels displayed a 5.5-fold increase in AIPC compared to CP (p = 0.005) and a 3.7-fold increase compared to PSC (p = 0.07). α-SMA mRNA levels in AIPC were 10.4-fold higher than in CP (p = 0.03) and 11.4-fold higher than in PSC (p = 0.04).

## Discussion

Despite progress in its clinical, radiological and morphological characterization in recent years, AIPC still remains an elusive disease. Its aetiology is unknown, its pathogenesis is only partly understood, and international consensus criteria for its diagnosis are still missing.[8] The fact that four different working groups have proposed their own diagnostic criteria [28]–[31]–although it may in part reflect heterogeneity of the disease in different parts of the world–clearly highlights the level of uncertainty in the approach to AIPC. Some recently published AIPC collectives [6], [24], [28], [32], [33] as well as numerous case reports have further improved the understanding of some aspects of the disease. Our patients' cohort represents one of the largest European single-centre series and in its clinical characteristics correlates well to the previously reported collection of cases.

Extra- and/or intrahepatic bile duct lesions histologically characterized by a dense infiltrate of IgG4-positive plasma cells have been reported in up to 74% of AIP-patients, [34] leading to the proposal of an entity called autoimmune IgG4-associated cholangitis [35] and questioning the existence of a previously reported association between AIP and primary sclerosing cholangitis. According to our data, AIP-associated biliary tract involvement, which was histologically proven in 64% of our cases (15% extrapancreatic), should not be regarded as a mere extrapancreatic manifestation of AIP, but rather as a subtype/sublocalization of a disease, which affects the pancreatic duct–usually in the pancreatic head–and may extend to the biliary system. More appropriately, this entity has been recently defined as autoimmune pancreatocholangitis (AIPC). [5] The AIPC-concept, which has been first suggested by Zen *et al*.,[36] relies on the embryological and morphological similarities between the ductal system of the pancreas and the biliary tract, and is also supported by the results of the present study, which demonstrate that the biliary tract involvement shares morphological and immunological characteristics with the pancreatic involvement itself and differs clearly from other biliary tract diseases, such as PSC.[37] Clinical aspects, such as patients' age (significantly higher in AIPC patients with extrapancreatic biliary involvement) and the consistent association of PSC with ulcerative colitis appeared to be of further help in distinguishing extrapancreatic AIPC from PSC.

Histopathology was the entry criterion used for the inclusion of AIPC cases in this study, according to previously described parameters,[24] whose validity could be confirmed also regarding the biliary tract manifestations of the disease. The similarity between the two subgroups of AIPC was further confirmed when the inflammatory infiltrate was examined in the different settings. In particular, the number of infiltrating B- and T-cells, macrophages and plasma cells was significantly higher compared to CP and, in the case of plasma cells, also compared to PSC. Another useful differential parameter was the number of CXCR5 and CXCL13-positive cells, which were found, although in small numbers, only in AIPC subgroups. CXCL13 and its receptor CXCR5 are involved in the organization and compartmentalization of lymph follicles and are overexpressed in various autoimmune diseases, like rheumatoid arthritis, Sjögren's syndrome, Hashimoto's thyreoiditis and Graves' disease, and in gut associated lymphoid tissue (GALT) of patients affected by ulcerative colitis.[20]–[23] This novel finding provides a further functional link between AIPC and some of the most frequently reported AIPC-associated conditions, such as Sjögren's syndrome and ulcerative colitis and gives further support to the still debated hypothesis of an autoimmune pathogenesis of AIPC. In analogy to mechanisms postulated in other autoimmune disorders [23], the formation of ectopic lymph follicles with germinal centers where B cells undergo maturation and activation, driven by the CXCL13-CXCR5 interaction, would therefore be essential for the pathogenesis of AIPC. Mature plasma cells would then synthesize large amount of immunoglobulins, which participate in tissue damage for instance by forming immune complexes.[33] The number of IgG4-positive plasma cells has been frequently reported as a useful tissue marker for the differential diagnosis between AIPC and other pancreatic/biliary diseases [6], [36], [38], [39] and for the differentiation of postulated morphological subtypes of AIP.[33] In the present study, the absolute number of IgG4-positive cells was higher in the AIPC group compared to both CP and PSC, although statistical significance was reached only in comparison with the PSC group. However, since the IgG4/plasma cell ratio was not different between groups, the observed increase of IgG4-positive cells in AIPC appears to be simply due to an increase in the total number of infiltrating plasma cells.

The analysis of the stromal reaction indicated substantial qualitative and quantitative differences in the composition and distribution of the fibrosis. The stromal reaction was mainly interlobular and periductal in AIPC and predominantly interlobular in CP. Moreover, the increased expression of collagen V–a collagen type that has been shown to accumulate in malignant disease [40]–and the more diffuse distribution of Tenascin C–an ECM protein that is overexpressed in pancreatic cancer [41]–and of αSMA in AIPC compared to CP may account for the radiological and macroscopic tumor-like appearance of AIPC. In addition, the stroma composition could reflect distinct underlying mechanisms for the development of fibrosis in CP and AIPC with a preferential activation of pancreatic stellate cells, which express αSMA, Tenascin C [41], [42] and collagen V (unpublished observation), in AIPC.

In conclusion, our clinical, morphological and immunological data support the existence of a possibly autoimmune-mediated inflammatory disease of the pancreato-biliary system of unknown cause, so-called autoimmune pancreatocholangitis (AIPC). AIPC usually affects the pancreatic head and may extend to other parts of the pancreas, as well as to the biliary tract. Morphological parameters are the only reliable criteria for the diagnosis, whereas the role of serum and/or tissue IgG4 and of CXCR5/CXCL13 as possible disease markers warrants further investigation.
